# My smart home: an auto-ethnography of learning to live with smart technologies

**DOI:** 10.1007/s00779-023-01725-0

**Published:** 2023-04-15

**Authors:** Line Kryger Aagaard, Toke Haunstrup Christensen, Kirsten Gram-Hanssen

**Affiliations:** Department of the Built Environment, Aalborg University, Aalborg, Denmark

**Keywords:** Smart home technology, Smart car, Auto-ethnography, Theories of practice, Learning theory

## Abstract

Smart home technology is expected to be widespread in the future and to accommodate a green transition to reduce and time-shift energy consumption. However, smart technologies also have social consequences, which are important to understand. At a basic level, we need to know more about learning to live with these technologies and how they influence our everyday practices and routines. Providing in-depth longitudinal insights into these processes, this paper presents an auto-ethnography of living with smart home technology: a 20-month diary kept by one of the authors. The paper uses theories of practice to investigate details of learning processes when interacting with three selected technologies: smart alarm and lighting management, smart control of heating, and a smart electric vehicle (EV). Theories of learning have a well-established tradition within theories of practice, and the concept of “knowing how to go on” and the concept of practical intelligibility are central in this work. This paper investigates the adoption of new smart technologies and how they interact with learning processes in different material and social contexts. Such an approach can lay the groundwork for further empirical research with a broader set of materials. It can also provide knowledge to assist in the design of better technologies and in developing policies and regulations to promote this.

## **Introduction**

Smart home technology (SHT) has been envisioned as a near future for a long time, although without any real breakthrough in the mainstream market to promote the technologies [1]. With a strong technology push from industry and with policy support, which sees SHT as a combined driver of economic growth and a means for efficient energy management, evidence of an expanding market for SHT is building [2, 3]. The perspective for market growth seems clear, according to sales figures; however, based on the way Big Tech companies approach the market, the evidence for actual energy savings related to SHT is uncertain, with some researchers warning against expanding energy consumption due to new features of comfort and convenience [4–8]. Further, research shows how the introduction of SHT will have consequences for the social organization in households with possible new roles developing between partners and generations [9–11]. Thus, with the spread of SHT into homes and everyday life, new questions arise about how people interact with the new devices, and questions around interaction with technologies can take different directions. In this paper, we explore how learning takes place when SHTs are integrated into everyday life, as practices related to existing tasks are changing, and new tasks are being introduced.

Previous SHT research has been interested in aspects of learning. A qualitative study of households living with Nest smart thermostats (including machine learning) concluded that the thermostats had difficulties responding to occupants’ sensed behavior, and people had difficulty understanding how the thermostats worked [6]. As a result, people performed workarounds to make the thermostats do what they wanted them to. This could include people wanting the thermostats to “forget” things they had “learned” previously but which were exceptions from regular patterns. Yang and Newman [6] suggest that smart technologies should help users gain a practical understanding of how the technology works. Thus, as part of ongoing interactions with users, the system should explain how it manages the input it receives rather than assuming that users are passive and in favor of letting the system handle everything. Another qualitative study, with less tech-savvy users, concluded that it is demanding and time-consuming to learn to use a smart home, and little help is available for this learning process [5].

A study with students in a real-life experimental setup concluded that, without specific training in the functionalities of SHT, even tech-ready and positive students did not use complicated functions which would have required them to invest time and effort to learn [12]. A survey of 72 householders with some form of SHT reported that 82% had never tried to write automation rules or do end-user programming, mainly because of a lack of competence and fear of accidently disrupting the system [13]. The same study reported that 37% of users experienced problems related to network failure or power outage. Such problems included feelings of insecurity when away from home or being notified about failures without knowing if they were caused by intruders or technical breakdown [13].

Many of these studies include SHT purchased by the households themselves; however, more future new and retrofitted buildings will include built-in smart systems for controlling energy and indoor climate. As Baborska-Narożny and Stevenson [14] established, these systems have a growing impact on building energy performance, and their malfunctioning assumes a large part in explaining performance gaps in terms of higher-than-predicted energy consumption. Energy efficiency is thus linked to residents’ use/non-use and learning related to the devices. One suggestion for improving technology is to perform usability tests on smart systems, to establish the extent to which users and managers understand the system and use feedback about poor usability to improve future design [14].

Several studies have pointed out that engagement and learning shift and develop over time [5, 15], making longitudinal studies of households relevant. Oliveira et al. [16] conducted a study in which household members were interviewed before and revisited 1 year after SHT installation. The study concluded that some initial concerns disappeared, especially in households with varied routines, and households living in large, partly occupied homes obtained added valued from the SHT. However, studies concluded that it took time and effort to configure the SHT, and, because of this, some participants deliberately used the simple features of the smart systems only [16]. A living-lab longitudinal study of a large number of households (*n* = 100) with smart heat control found that preferences for temperature settings, energy savings, and time spent on controlling the technology varied [17]. Learning processes were discussed in relation to householders’ knowledge, or lack of knowledge, about how much energy or money could be saved from heating less. The thermodynamics of home heating are complicated, and few people fully understand them. Several householders in the study preferred not to invest too much time in the technology, instead being prepared to pay for the convenience of not adjusting the controls too much [17].

Similar findings were presented by Jacobi et al. [18] who also conducted a living lab study in which participants (14 households) were interviewed prior, during, and after an 18-month period with SHT installed. Mapping barriers related to SHT appropriation, the study also pointed towards the importance of “contextual detail” [18, p. 1620] and “practices, routines and use cases” [18, p. 1628] in understanding the difficulties that users experience with SHT.

Based on the studies presented above, learning to live with SHT is not a straightforward process; it takes time and requires effort from householders to learn. The learning process, however, also relates to usability of the actual technology. To conduct further research into how learning processes related to SHT unfold, a vocabulary and a theoretical approach are needed to gauge what this learning might include. The purpose of this paper is to develop such an approach, to study what happens as people learn to live with new smart technologies in the home.

Bodily routines and emotional responses are important parts of learning processes; however, these can be difficult to grasp with conventional methods such as interviews and surveys comprising most existing research. As some of the above mentioned studies demonstrate, living labs represent a useful approach in providing long-term perspectives. With a similar interest, the present paper provides longitudinal chronological insights, however, with an alternative approach allowing for a detailed insider perspective—an auto-ethnography based on the diary written by one of the authors during 20 months. With this material, the paper presents an in situ narrative of smart home living and learning.

The following sections will first describe our theoretical approach to learning based on theories of practice. Next, the methods of our auto-ethnographic study are described. Studying a single case in detail cannot provide general knowledge about a whole population or about variations in practices in different types of homes. Rather, the auto-ethnographic approach used in this study will provide the basis for a detailed assessment of the complexity and dynamic nature of the learning processes related to SHT. After sections 2 and 3, an analysis of the results is presented, followed by the conclusions drawn.

## Theory

Following Schatzki, a social practice can be defined as a bundle of activities, i.e., “an organized nexus of actions” [19, p. 71] where actions are understood as bodily doings and sayings. According to Schatzki, these nexuses of actions are linked together through (1) practical understandings (e.g., knowing how to do something), (2) rules (e.g., principles or instructions), (3) teleo-affective structures (e.g., the goal of practices or normativized emotions), and (4) general understandings (e.g., religious beliefs) [19, p. 77]. Other authors have proposed other conceptualizations of the links keeping the nexus of actions-in-practice together [e.g., 20, 21].

The concept of “practical intelligibility” plays a key role for Schatzki in understanding how individuals perform practices: “Practical intelligibility is what makes sense to a person to do. It governs action by specifying what an actor does next in the continuous flow of activity” [17, p. 74-75]. Practical intelligibility is closely related to the linking element of practical understandings, in that practical understanding “executes the actions that practical intelligibility singles out” [19, p. 79] in a given situation. One might understand this as practical intelligibility providing the impetus and “directionality” for the actions of individual practice performers, but it is through practical understandings—the “knowing how X” [19, p. 77]—that these actions are carried out. Schatzki further describes practical intelligibility as “itself determined by mental conditions, many of which (are) formed during the process of learning and being trained and instructed to carry on the practices involved” [19, p. 81].

A key concept for Schatzki is Wittgenstein’s concept of “knowing how to go on” [22] in relation to how learning takes place, which is another way of expressing what practical intelligibility is. Learning is central to the performance of practices, as, while practices must be learned, what is learned is not “the practice as such but how to go on in them” [22, p. 34]. In other words, through acts of learning, the practical intelligibility of “the learner” is elaborated and extended with the outcome being augmented operability in the sense that the learner attains greater facility in performing practices. However, augmented operability can be achieved in ways other than learning, for instance, by applying new tools that enable the practitioner to perform practices with a higher facility and excellence. In this way (augmented), operability can be seen as distributed between the embodied practical intelligibility and attributes of the environment in which the practitioner is embedded, e.g., the material arrangements.

While social practices are collective entities—“practices-as-entities”—the performance of practices and the related practical intelligibility happen on the level of individuals. Together, with the understanding of practical intelligibility as the key to the performance of practices and with the understanding of it as a characteristic of the individual, practical intelligibility becomes an important mediator between the collective practices and their individual performance. When combined with practice arrangements, collective practices shape practical intelligibility through learning and the biographies of the individual practitioner. Thus, practical intelligibility transgresses the classic dichotomy between the actor and the structure. As Schatzki [22] points out, learning is always situated within practices but also assumes the shape of progressive learning through individual learning paths that take place across space, time, and practices. Thus, one might acquire certain skills within one practice (e.g., programming skills learned as part of formal education) which might later be integrated into the performance of other practices one is engaged in performing (e.g., programming SHT in everyday life). Similar to such learning biographies of individuals, Dreier [23] describes individual learning sequences taking place across social contexts and localities.

The material environments play a role both in the performance of practices and in the learning of “how to go on.” To Schatzki, “materiality helps compose sociality and social phenomena” [24, p. 133], but he rejects the idea that materiality is a linking element of practices per se. In contrast, other social practice theory approaches have adopted material elements, such as technologies, among the constituting elements of practices [20, 21]. With a slightly different approach, Schatzki refers to practice arrangements to which material arrangements belong. In this, material arrangements are understood as complexes of individually connected material entities, including humans, human-made artifacts, living organisms, and non-human made things. Kemmis et al. [25] have developed a similar concept within a learning-theoretical context by conceptualizing practice architectures. This perspective follows Schatzki’s [19] site ontological approach by studying “how in practice, in *this* specific site, *this* practice and *these arrangements* come to assume *this* distinctive shape and form” [26, p. 1178, emphasis in original]. Practice architectures distinguish between cultural-discursive, material-economic, and social-political arrangements.

In studying learning in relation to specific practices, it is possible to identify different types of learning forms as these unfold empirically when specific practices occur within specific sites. For instance, Christensen’s [27] study identifies three different types of learning related to Do-It-Yourself (DIY) work in Danish homes. Here, learning to become a (better or more skilled) DIY practitioner involved (1) searching and acquiring knowledge on “how to X” via advice from friends, family, colleagues, websites, or from professionals; (2) learning through concrete collaboration with friends or family members helping with DIY tasks (similar to the situated learning of Lave and Wenger [28]); and 3) learning via interaction with materials.

Thus, a practice theoretical approach to the study of learning to live with SHT should focus on how augmented operability can be achieved through learning that enhances the practical intelligibility of the practitioner. As part of this approach, it is relevant to discuss whether SHT, as a form of materiality and part of the material arrangements of the home, differs from other types of material entities in relation to how learning takes place and whether there is variation in different types of SHT. Learning could be initiated by different situations, actions, or teleo-affective goals, and we will pay attention to these differences in the present paper.

## Method

The paper builds on an auto-ethnographic diary written by one of the authors over the course of 20 months. Auto-ethnography was developed as a method in the 1980s as part of a critique of traditional ethnographic studies, also known as the crisis of representation, that questioned established relations between subject and object [29]. Auto-ethnography includes a broad range of approaches such as personal narratives, first person accounts, experimental ethnography, confessional tales, and indigenous ethnography [30]. Also, auto-ethnography positions the writer with the identity of being a researcher and a personal self at the same time, implying a position from which to study their own everyday life [29].

As auto-ethnography questions established relations between subjectivity and objectivity, the language of the method is often different from more conventional types of academic writing. Text is often written in the first person and focuses on single cases, taking the form of literary writing, including emotional and bodily experiences. It has been argued that auto-ethnography is especially relevant when studying sensuous and bodily phenomena, such as embodied skills and habitual enactments, and learning new practices, which can be difficult to verbalize and thus difficult to study with methods such as qualitative interviews [31]. The auto-ethnographic approach was chosen for this reason and also as a way to provide more in-depth insights and add a day-to-day longitudinal perspective on living with SHT and the related challenges, thus providing different insights than studies building on surveys, questionnaires, or interviews.

The strength of auto-ethnography is, among others, its ability to “document experiences that often go untold in everyday life and communication research. (…) (It) seeks to reveal unseen communicative practices and cultural constructions for purposes such as describing and sensitizing.” [32, p. 75]. The auto-ethnographic narrative challenges the view of the researcher as neutral or distanced and instead brings attention to their active engagement. In this, the auto-ethnographic writing process can be considered a learning experience in itself [33, p. 183].

As such, the auto-ethnographic material of the paper is written to include both bodily sensations and emotional responses which are of high analytical significance, also to a practice theoretical approach which the paper takes, as described in the previous section. Another strength of auto-ethnography vis-a-vis other methods is its ability to “respond to the fluctuating patterns of relationships that exist within and extends beyond a house” [10, p. 10]. As the paper will reveal, these relationships are indeed central to the various learning processes depicted in the auto-ethnography.

It has been argued that auto-ethnography, together with ethnography of silence and of infrastructures, is especially relevant when studying SHT [10]. One argument suggests that we only notice technologies when they fail or when they do not work as expected, that the flow of data behind the technologies is largely invisible, and that methods are needed that comply with these eventualities. A further argument for applying auto-ethnography in the study of SHT concerns relations between technology and users developing over time and is therefore difficult to grasp with methods involving short-duration presence with the technologies, compared to what is possible in one’s own home [10]. This being said, Hine [10] notes that the strength of auto-ethnography is not necessarily about taking notes exactly when things happen, but rather about writing reflections on what has happened or noting expectations about what will happen.

Based on this discussion it can be argued that auto-ethnography has advantages in the study of learning to use SHT. However, there are also limitations which include that only one perspective and case are studied, and a risk of self-indulgence could lead to suggestions of combining auto-ethnography with other methods [31]. In this paper, we conduct in-depth analysis of just one auto-ethnographic study, primarily to develop concepts and understandings rather than to make a broader or more representative analysis. The paper is part of a larger research project called eCAPE (see details in the Funding section) that draws on other types of qualitative methods and statistical data to allow for broader, more generalizable knowledge on SHT. Furthermore, not only the auto-ethnographer but also two other authors were involved in the writing and analysis of this paper. This allows for a combination of the insider perspective and an external view of the process of learning to live with SHT, giving room for multivocal analysis and interpretation rather than being restricted to “a very singular personal perspective” [10, p. 10] which is one of the common critiques of auto-ethnography. The paper is thus not written in the first-person, except when quotations from the diary notes are included. Instead, we use the first name of the author, Kirsten, who is the author of the diary and owner of the home.

The diary writing begins one and a half years after the initial installation of a smart alarm which follows from an unpleasant experience with a burglary. Shortly after the alarm is installed, Kirsten is writing a research proposal including questions about SHT. Using her own experience with the alarm seems to be an obvious opportunity for further research. After receiving the research grant, Kirsten finds herself in a situation of needing to install more smart technologies to enable undertaking the auto-ethnography, and thus, in agreement with her husband, she invests in smart control of heating and lighting. Later, when the couple’s old car breaks down, they buy a smart electric car (EV). This purchase does not come from Kirsten’s research interest in smart technologies, and it can be questioned whether a car should be considered as part of a smart home. Nonetheless, the car is included in the diary notes since both processes of learning and reflection on the car and the home are related. The diary writing spans approximately 20 months, following the installation of the first smart control for lighting and heating and, later, the smart EV. Table 1 shows the process of acquiring the technologies and the writing of the dairy. The diary is written in Danish, and the authors have translated the extracts used in this paper.

**Table 1:** Timeline of diary writing

Time	Incidents or themes in diary notes	Page in diary
Spring 2017	Burglary and installation of smart alarm	
Summer 2017	Living with smart alarm and writing research proposal	
November 2018	Research project begins, and diary writing is initiated by summing up what has happened in relation to SHT before diary initiation	1
December 2018	Ordering smart light switches on 15th of December Ordering smart heating system on 20th of December Installing smart light switches on 26th of December	3
January 2019	Struggling with programming smart lights Installing smart heating system on 7th of January	8
March 2019	Having familiarized with smart lighting and learned to program	15
May 2019	Sharing the diary with research group and discussing it	18
Summer 2019	Learning about how changes in seasons, weather, and sunlight throughout the year impact on technology use	20
Fall and winter 2019	Experiencing different technical problems, solving them, and simply using the technologies	23
March 2020	Purchasing the EV	31
September 2020	End of diary	44

## Analysis

The story of learning with SHT has several chapters. The smart home diary begins with an unpleasant incident: a series of thefts in the home culminate with a burglar breaking in and entering Kirsten’s house while she and her husband are asleep. Kirsten catches a glimpse of a flashlight on the stairs outside their bedroom, and, although the burglar flees before getting away with anything, the experience leaves Kirsten and her husband in shock. Something needs to be done.

The following diary note explains the episode and the beginning of the diary:

The “choice” of the alarm took place (as far as I remember) by googling the alarm and we got hold of one of the largest companies that deliver that kind. They could send a man out within a few days, which was important to us. We needed to know that we were doing something and were not just victims who could be exposed again. When he arrived, it turned out that the type of alarm they were selling was a new "smart" alarm which includes wireless cameras with motion sensors in several rooms, as well as sensors on relevant exterior doors, the ability to zone the house depending on whether it is night and we are sleeping in the house, needing to be able to go to the toilet without activating the alarm, or whether we are not at home. The electrician who, at the same time, installed our outdoor lighting, said that he could also sell us an alarm, although he actually thought the other company had a better product. So, we bought this (from the other company) and got it installed soon after. Or, in fact, you do not buy it, you pay a monthly rent for the equipment and for the security service that follows if the alarm goes off. In the time before and after installation, I was working on an article in collaboration with an international colleague about SHT, so the fact that I had suddenly come to live with something like this myself started several reflections about researching this type of technology while, at the same time, living with it. I would not say that we deliberately chose to have SHT in our home, rather that it was something happening because we had to do something about the several burglaries and especially the last one while we were at home. Many of our neighbors on the street have similar technologies, so it is also part of a normalization that caused us to get it. When everyone else has an alarm, we become the obvious house to break into if we did not get one too. So, I ended up a little unintentionally in the situation that I became the owner of something I wrote about at the same time–but the idea of ​​combining the two approaches seemed obvious. (p. 2)

This chain of events is what initiates Kirsten’s smart home, marking the beginning of a complex journey of learning. Based on the theory presented previously, what we are looking for in these notes relates to understanding when Kirsten is experiencing her practical intelligibility as enhanced, following from learning and knowing how to go on with the new technologies and seeing what initiates this.

The wish to feel safe at home, to take action, and to avoid future break-ins sets off the change in the material arrangements, i.e., the installation of the alarm. This change in the material arrangements happens within a social context where alarms are normalized: all neighbours have them. Later, when Kirsten receives the research grant in which she has included auto-ethnography writing, she finds herself in a situation where she must upgrade her home with more smart features to have something to include in her diary. This second phase in the making of her smart home is thus initiated by scholarly interest. She and, to some extent, her husband install a smart heating system and add smart control of lighting to the alarm system. Finally, in the third phase, the couple acquires a smart EV. This was not initiated by scholarly interest but resulted from the breakdown of their old car. An environmental interest directed their wish for an EV, followed by browsing the market and knowing what was possible to acquire at the time of the breakdown of the old car. The telos in this third phase relates to a wish for sustainable car driving, and the EV, apart from being electric, includes features such as being connected to the phone via apps and Bluetooth, connected to an electric charging system involving software (thus needing updates), and smart features as part of operating the car, such as digital voice assistance.

The particular materiality of SHT needs to be considered in studying what goes on in detail when people learn to live with new smart technologies in their homes. A particular SHT characteristic is its connectivity; smart technologies are connected to the internet and can be accessed remotely. As such, the materiality of SHT stretches across time and place, beyond its immediate physical appearance. Behind the technology, a whole (partly black-boxed) infrastructure resides. The particular materiality of different SHTs in Kirsten’s smart home is comprised by a number of components. For instance, the smart alarm not only consists of a physical device but also includes software, and furthermore, it is connected to a security service and customer support. This underlying “infrastructure” characterizing SHT enables different forms of interaction between the individual (practitioner) and the technologies which influence the process of learning.

As described in section 2, the practice theory perspective regards the notion of practical intelligibility as key in processes of learning. Practical intelligibility is shaped by mental conditions formed during learning processes. Through learning, practical intelligibility is expanded which gives directionality to the individual practitioner, making her know how to go on. The outcome of learning is augmented operability. How does one achieve this in the process of making one’s home smart? The three parts of Kirsten’s smart home (the alarm and lighting system, the smart heating, and the EV) each give rise to different scenarios, challenges, and processes of learning. The following analysis goes into detail about each of the systems and how Kirsten’s journey of learning takes shape.

### The smart alarm system

The materiality of the smart alarm consists of several components: cameras and sensors rented from the security company with a monthly subscription, an app to monitor and manage the activity of the alarm and lighting, key chips to unlock the system, smart lighting bulbs and switches programmed for activation and de-activation, and the alarm, based on timed schedules or controlled via the app. Part of the material arrangement includes the software embedded in the system, the video and motion recordings, and the security and customer support function, implying that the material arrangements stretch beyond the home. A question arising is whether this component should be counted as part of the alarm system’s materiality or its social embeddedness.

Kirsten and her husband need to familiarize themselves with these new technologies, learn how to use them, make settings, and integrate them into their daily lives. Kirsten learns “how to go on” with the smart alarm in several ways. First of all, learning occurs via interaction with the materiality. An example is the so-called “rule making.” Here, Kirsten codes scenarios into the app, e.g., making the lights turn on in accordance with the alarm or setting the lights to turn off when the night alarm is turned on. She tests that the connection between the lights and the alarm is working by turning it on and off, confirming the rule.

Another way to learn is when errors occur. For instance, there is one point early in the diary where the lights are not turned on when Kirsten returns home. She finds out why the rule does not work by phoning the customer support; she needs to add an end date; otherwise, the rule only works for 1 day. This information causes Kirsten frustration, but she also realizes that she needs to be patient and believe in herself and believe that she can make the lights work in the right way:

Then I called the company and asked why my smart rules did not work. I was told that they had worked and then had stopped. She (I think it’s the same person I've talked to twice before) looked at my rules and told me that they were set to work for only one day because I did not have an end date… Yark!!! Am I really so useless for doing such programming? Now I have to get back on track again and try to believe that I can make the lights work. (p. 11)

In this way, the diary includes examples of “learning how to learn.” Thus, the augmented operability can include more general enhancements in the practitioner’s capability to learn new ways of doing. These capabilities might have relevance across various forms of SHT and can in this way be seen as transferable skills. Furthermore, the example illustrates how the telos can include the will to succeed in learning new skills.

Another example of learning through incidents of error is a disconnection in the system: Kirsten cannot make her phone and the system connect. She therefore phones customer support:

Well, I called, and it turned out to be as banal as the z-wave extender I have had “fallen out”. I was guided to unplug it, remove the z-wave device, wait a bit, and then reassemble it. The man at the other end can then apparently see in his system if my z-wave has been properly inserted. (p. 23)

Following this scenario, Kirsten needs to remake the rules for their lighting and discovers that by now, she understands the logic and finds it easy to do the programming. She has now learned to make rules and the logic behind the system.

What characterizes the SHT is, among other things, the remote access as seen in the scenarios above. This remote access influences how and when learning can take place. Another example of this occurs one day when the technology is not working properly: the camera records even though the alarm is off. Kirsten is abroad, and her husband discovers that one of the cameras blinks while he is home, indicating that it is recording, and he phones customer support. A technician solves the problem by updating the system and finding the error. Everything is done at a distance, meaning that the very materiality of the technology (its connectivity and way of being remotely accessible) obviates the need to always learn yourself; the customer support can resolve the issues.

Learning can also be spurred by unexpected implications of adapting new practices which might conflict with established practices and norms. One example of this is the new possibilities of home surveillance, following the installation of the alarm, which were not part of the initial purpose of acquiring it. The alarm system app allows one to see when people enter and leave the home, and Kirsten is, for instance, able to see when her husband goes to bed and wakes up if she is not at home. She does this one time while being away at a conference, checking if he is awake before calling him, to avoid waking him up if he is asleep. With this arises an ethical dilemma of surveillance, also related to the house cleaner:

(…) I could see when our house cleaner came and went. Something I checked a few times but always had a bad conscience about doing, because we are really happy with her work and have absolutely no reason to check on her… (p. 3)

Kirsten learns about the new possibilities of the alarm, and this raises new questions for her newly adopted home safeguarding practice of remote monitoring. However, the telos associated with the home monitoring practice collides with established norms of privacy and understandings of interpersonal relations as governed by mutual trust rather than surveillance and control. The example shows how such conflicting teloses can initiate new reflections that eventually affect the performance of practices through, e.g., refraining from applying newly enhanced operabilities. Over time, the ethical dilemmas related to surveillance, however, became less significant for Kirsten, according to the diary. For instance, Kirsten later writes about checking whether the house cleaner had been there during their holiday, and how she felt happy to discover that the house cleaner had been there a full day (meaning that the house cleaner had probably taken the time to do a thorough cleaning). This indicates that the ethical dilemma seemed to vanish when the purpose of surveillance felt justified. Finally, when Kirsten’s operability is augmented, she gets new ideas about how she and her husband could improve the smart functions they have in their home. For instance, she would like the system to include the possibility that the lights not only follow specific times of the day but also the changing solar times that can be found online. This is a capability that the product does not yet include, and Kirsten is frustrated about this. This situation illustrates the different stages in learning: first, learning about the basic functions of the technology and then learning enough to imagine further developments of new possibilities.

### The smart heating

The materiality of the smart heating consists of eight thermostats running on battery, one control display placed in the home office, and apps on a phone (Kirsten’s) and tablet (her husband’s). In this case, no customer support is available.

Learning biographies play a role here as Kirsten has gained know-how and competences through her previous experience with the smart alarm. For instance, through prior learning experience with the smart switches of the alarm system, Kirsten’s operability is augmented, and she knows (i.e., through practical intelligibility) that they need to consider the distance and range of the signal when they place the control panel so that it is not located too far from the thermostats, as this caused problems with the lighting system.

As with the smart alarm system, Kirsten learns through interacting with the materiality, making settings in the app, and she talks to friends, family, and colleagues. Often, a mix of different learning forms occurs in which Kirsten sets out to make settings without a guide, giving it a try by interacting with the materiality, and then when coming to a halt, studying the guide.

The diary includes several examples of the technology acting unpredictably or unintentionally which confuses Kirsten. These examples illustrate how the black-boxing of technology makes her refrain from learning. The following scenario is illustrative:

Very strange. Today at 11 o’clock, I started to think that it was a bit cold, and then I looked at my app and could see that it was set to ‘not at home’. The first reaction was to press ‘at home’ on the in-home display, but then I pulled myself together and looked around a bit in the system to see what the circadian rhythm was set up for, and it turned out that it was programmed to ‘not-home’ during daytime on Thursdays and Fridays. It fits with it also being a Thursday when our son stopped by and it was cold. But it is very strange if, for two months now, we should have lived with the heat turned off every Thursday and Friday without having noticed. [*This excerpt was written during a COVID-19 lockdown, when Kirsten and her husband were working from home.*] Conversely, it is strange if the system itself at some point has changed the circadian rhythm on its own. (p. 35)

Kirsten reflects about the agency of the technology, if “the system itself changed the circadian rhythm.” She does not really believe so; however, as she writes, she will probably never find out what happened. This exemplifies that lack of transparency of how such a system works in the end might obstruct learning and augmented operability.

### The smart car

What can be seen as the third phase of Kirsten’s smart home is the purchase of a new car, an electric vehicle (EV). She and her husband purchase the new car since their old one has broken down, and they wish to drive more sustainably. The materiality includes the car (including a display screen), car software (that needs to be updated continuously), an app, the charging system at home and around, and key cards.

As with the previous two cases learning takes place through interaction with the materiality. In this case, Kirsten explores the display screen from which the car’s different features are operated, such as the mirrors and the buttons on the steering wheel. Learning also takes place through guidance, such as videos in the display screen, and thus, guidance is integrated into the materiality of the car.

Furthermore, learning takes place through others, such as Kirsten’s sons. One of them shows a particular interest, prepared to experiment, and looking things up more than Kirsten and her husband. This exemplifies the importance of different learning biographies. In this scenario, learning is shared by family members; Kirsten feels good and safe about sharing the responsibility of getting to know the car. However, her motivation for learning at times decreases as other family members take responsibility for (and interest in) learning about it to a higher degree. For instance, Kirsten’s husband takes the main responsibility for charging the car. He is interested in charging when pricing is low, as this means that there is more renewable energy produced at these times. The fact that Kirsten’s husband takes on this responsibility means that Kirsten often forgets about charging, and even though she is interested in sustainable consumption, she is not as active in this as her husband:

I still have not downloaded the app that allows me to track our consumption and to see when energy prices are low, although I am in principle interested in it. (p. 37)

Interaction with the smart car also illustrates how learning takes place through barriers or incidents, e.g., when the family learns about security gaps in the system. One of the family members enters a wrong password for the car three times causing the whole family to be prevented from using the app until a new password is created. Creating a new password is only possible through Kirsten’s husband’s e-mail account, as the car can be connected to one email account only. Thus, the family learns about the vulnerabilities of the car. From now on, they will always bring a key card with them and not rely only on the app on their phones when they drive the car, if the app should not work. As such, the practical intelligibility and understandings related to the practice of EV driving have been moderated.

The remote access that smart technologies enable plays a role in the case of the EV. For instance, by tracking her son’s route through the app, Kirsten learns remotely about the capacity of the car’s battery. By learning in this way, as well as by driving the car herself, she realises that planned trips are no problem battery-wise, but longer spontaneous trips can cause problems. Again, the tracking of other family members gives rise to moral reflections on surveillance, especially in the beginning, but appeared to be more routinised later. Checking if her husband is on his way home, or him checking her, as part of deciding when dinner can be served, is not considered an ethical dilemma later on in the writings.

## Conclusion

This paper provides a narrative of the learning processes involved when living with SHT. Drawing on theories of practice, a focus has been placed on the practical intelligibility and augmented operability in processes of learning: how does one “know how to go on” and how does one learn and refrain from learning, when not knowing how to go on? Auto-ethnographic material from a 20-month diary of one of the authors enabled insight into everyday scenarios, thoughts, and frustrations that arise when smart technologies move into the home. The material provided a tale of everyday life, family relations, the challenges arising when learning is difficult, and the rewards that follow when learning is achieved. Learning is a central component in the domestication of SHT, and it shows how the implementation of these technologies is a dynamic process that includes progress as well as blockages, confusion, and (un)certainty. With the auto-ethnographic approach, the paper demonstrates how this method “can usefully pay a close reflexive attention to the varied textures of lived experience with digital technologies” [10, p. 31].

When it comes to what initiates acts of learning, we found that, in addition to “not knowing how to go on,” other things trigger new processes of learning, including, importantly, situations where people adopt a new aim for learning to do something new, such as deciding to install a security system to realize the project (telos) of (re)establishing a safe home. Secondly, interactions with others may introduce one to new ideas or possibilities to be adopted as a new goal or project. Thirdly, the analysis shows how the augmented operability that follows from learning to do new things can spur new ideas on what to learn next. This can also cause conflicts with other practices and as such initiate new learning, as was the case with the possibilities of monitoring others remotely (and augmented operability enabled by the materiality of the alarm system and the EV).

With regard to how learning unfolds, the auto-ethnographic study points to three clusters of ways through which learning happens: firstly, learning happens in many cases via interaction with the material—often assuming the shape of “trial-and-error” interactions when trying out something new (e.g., setting up a programme). The materiality often reacts in unexpected ways which leads to trying out new ways of interacting with it. Secondly, customer services, either personal phone support or digital guidance (e.g., video tutorials), play a key role in many learning situations. Thirdly, in several cases, learning happens through social relations (i.e., interaction with others), in some cases similar to what Lave and Wenger [28] term “situated learning.” In addition, learning biographies of individuals play an important role in shaping the learning processes, e.g., in the case of previously learned skills that are transferable to new learning incidents.

Concerning what is achieved through learning processes with SHT, i.e., the achievement of augmented operability through extending the practical intelligibility of the practitioner, we found several types of new capabilities that are acquired, such as improving programming skills (e.g., setting schedules for control of lighting) which in Kirsten’s case could be part of expanding an already existing practical intelligibility acquired previously. We also found several examples of new skills or competences acquired that could be closely related to the characteristics of the technologies and systems being interacted with (e.g., how to programme the lights or the Z-wave connection problems). One might wonder to what extent these examples of augmented operability are intimately locked into the specific technologies at hand, or whether such competences, skills, and “ways of doing” (i.e., practical intelligibility) have a more general character capable of being transferred across different SHTs and their related practices. In the present case, there are examples both of how learning is taken from one system to another, e.g., how far the Z-wave can reach, and of cases that are completely different, e.g., the different rule making systems. In other words, does the practitioner through acts of learning develop a more general practical intelligibility that will help incorporate other SHTs into everyday practices? Or will the practitioner need to “start from scratch” with each new SHT because they differ significantly from each other in terms of material design features and levels of transparency? If the latter is the case, this might comprise a more fundamental challenge to the uptake of SHTs in people’s everyday life.

The auto-ethnography material utilized in this paper ended in 2020, before the energy crisis in 2022 in which SHT, to an even higher degree, can be seen as relevant in managing energy consumption. To be noted, Kirsten installed an app for monitoring the varying energy prices in 2022 (like most other Danish householders did that year). Functionalities of the smart heating control, including zoning the house and only heating parts of the home during different times of the day and week, were taken up during the fall and winter of 2022 in Kirsten’s home. This indicates that SHT use and propagation are likely to expand also as a consequence of the energy situation, which points towards further relevance of studying and enhancing the usability and accessibility of living with SHT.

The main aim of this paper was to develop a language to understand the learning process taking shape when new smart technologies enter into everyday life. Building on theories of practice and auto-ethnographic material from one of the authors, we have developed contributions to such a vocabulary. Key concepts are augmented operability (the outcome of learning in the form of the performance of new—or changed—practices, e.g., time-shifting energy consumption), practical intelligibility (the capabilities of the practitioner in performing such new or changed practices), and learning biographies (the historical shaping of the individual practitioner’s practical intelligibility, which shapes one’s learning trajectory when facing new smart technologies). These concepts establish a theoretical framework that might help bring processes of learning to the fore in SHT studies and, by doing so, provide a better understanding of the domestication of SHT.

Future studies on learning processes related to how people learn to use new smart technology (with a wider type of participants than included here) may lay the groundwork for better understanding human-technology interactions. This can improve the development of technologies that allow people to learn to live in a smart home.

## Data Availability

The auto-ethnographic diary will be made publicly accessible after the publication of the paper.

## References

[1] Hui TKL, Sherratt RS, Sánchez DD (2017). Major requirements for building smart homes in smart cities based on Internet of Things technologies. Future Gener Comput Syst.

[2] Sovacool BK, Furszyfer Del Rio DD (2020). Smart home technologies in Europe: a critical review of concepts, benefits, risks and policies. Renew Sustain Energy Rev.

[3] Wilson C, Hargreaves T, Hauxwell-Baldwin R (2017). Benefits and risks of smart home technologies. Energy Policy.

[4] Aagaard LK (2021). The meaning of convenience in smart home imaginaries: tech industry insights. Buildings and Cities.

[5] Hargreaves T, Wilson C, Hauxwell-Baldwin R (2018). Learning to live in a smart home. Build Res Inf.

[6] Yang R, Newman MW (2013). Learning from a learning thermostat: lessons for intelligent systems for the home. Proceedings of the 2013 ACM International Joint Conference on Pervasive and Ubiquitous Computing.

[7] Strengers Y, Hazas M, Nicholls L, Kjeldskov J, Skov MB (2020). Pursuing pleasance: interrogating energy-intensive visions for the smart home. Int J Hum Comput Stud.

[8] Strengers Y, Nicholls L (2017). Convenience and energy consumption in the smart home of the future: industry visions from Australia and beyond. Energy Res Soc Sci.

[9] Wilson C, Hargreaves T, Hauxwell-Baldwin R (2015). Smart homes and their users: a systematic analysis and key challenges. Pers Ubiquitous Comput.

[10] Hine C (2019). Strategies for reflexive ethnography in the smart home: autoethnography of silence and emotion. Sociology.

[11] Aagaard LK (2022) When smart technologies enter household practices: the gendered implications of digital housekeeping. Housing, Theory and Society 1–18. 10.1080/14036096.2022.2094460

[12] Wright D (2019). Smart home technology adoption and learning. 2019 IEEE International Professional Communication Conference (ProComm).

[13] He W, Martinez J, Padhi R, Zhang L, Ur B (2019). When smart devices are stupid: negative experiences using home smart devices. 2019 IEEE Security and Privacy Workshops (SPW).

[14] Baborska-Narożny M, Stevenson F (2019). Service controls interfaces in housing: usability and engagement tool development. Build Res Inf.

[15] Hargreaves T, Nye M, Burgess J (2013). Keeping energy visible? Exploring how householders interact with feedback from smart energy monitors in the longer term. Energy Policy.

[16] Oliveira L, Mitchell V, May A (2020). Smart home technology—comparing householder expectations at the point of installation with experiences 1 year later. Pers Ubiquitous Comput.

[17] Sovacool BK, Osborn J, Martiskainen M, Lipson M (2020). Testing smarter control and feedback with users: time, temperature and space in household heating preferences and practices in a Living Laboratory. Glob Environ Change.

[18] Jakobi T, Ogonowski C, Castelli N, Stevens G, Wulf V (2017). The catch(es) with smart home: experiences of a living lab field study. *Proceedings of the 2017 CHI Conference on Human Factors in Computing Systems*.

[19] Schatzki T (2002). The site of the social: a philosophical account of the constitution of social life and change.

[20] Gram-Hanssen K (2011). Understanding change and continuity in residential energy consumption. J Consum Cult.

[21] Shove E, Pantzar M (2005). Consumers, producers and practices: understanding the invention and reinvention of Nordic walking. J Consum Cult.

[22] Schatzki T, Grootenboer P, Edwards-Groves C, Choy S (2017). Practices and Learning. *Practice theory perspectives on pedagogy and education* (pp. 23–43).

[23] Dreier O (2007). *Psychotherapy in everyday life*.

[24] Schatzki T (2010). Materiality and social life. Nature and Culture.

[25] Kemmis S, Wilkinson J, Edwards-Groves C, Hardy I, Grootenboer P, Bristol L (2014) *Changing practices, changing education*, 2014th edn, Springer Singapore. 10.1007/978-981-4560-47-4

[26] Kaukko M, Wilkinson J (2020). ‘Learning how to go on’: refugee students and informal learning practices. Int J Incl Educ.

[27] Christensen TH (2016). Læring inden for hjemmet som praksis-arrangement: Gør-det-selv projekter, identitet og materialitet. Kognition & Paedagogik.

[28] Lave J, Wenger E (1991). *Situated learning: legitimate peripheral participation* (1st Edition edition).

[29] Butz D, Besio K (2009). Autoethnography. Geography Compass.

[30] Ellis C, Given LM (2008). Autoethnography. The SAGE Encyclopedia of Qualitative Research Methods.

[31] Larsen J (2014). Ethnographies and cycling. Int J Soc Res Methodol.

[32] Bolen DM, Allen IM (2017). Autoethnography. *The SAGE Encyclopedia of Communication Research Methods*.

[33] Collinson JA, Hockey J, McNamee IM (2005). Autoethnography: self-indulgence or rigorous methodology?. Philosophy and the Sciences of Exercise, Health and Sport: Critical Perspectives on Research Methods.

